# Supplementary material for: What makes music memorable? Relationships between acoustic musical features and music-evoked emotions and memories in older adults
Source: PLoS One. 2021 May 14;16(5):e0251692. doi: 10.1371/journal.pone.0251692 (PMC8121320; doi:10.1371/journal.pone.0251692)
Supplement: S1 Table — (PDF) [file pone.0251692.s001.pdf]

**S1 Table.** Full list of the 140 songs used in the study.

| Song genre | Artist | Song title |
|---|---|---|
| 1950s popular songs (30) | Pirkko Jaakkola | *Pariisin taivaan alla* |
| | Laila Kinnunen | *Lazzarella* |
| | Eila Pienimäki | *Vanhan veräjän luona* |
| | Kipparikvartetti | *Kaunis Veera* |
| | Olavi Virta | *Sokeripala* |
| | Juha Eirto | *Tiikerihai* |
| | Eero Väre | *Kultainen nuoruus* |
| | Lasse Liemola | *Anna pois* |
| | Doris Day | *Whatever Will Be, Will Be (Que Sera, Sera)* |
| | The Platters | *Smoke Gets in Your Eyes* |
| | Little Richard | *Long Tall Sally* |
| | Harry Belafonte | *Day-O (The Banana Boat Song)* |
| | Nat King Cole | *Quizás, quizás, quizás* |
| | Louis Armstrong | *Mack the Knife* |
| | Chris Barber | *Petite Fleur* |
| | Metro-tytöt | *Orvokkeja äidille* |
| | Helena Siltala | *Ranskalaiset korot* |
| | Annikki Tähti | *Kuningaskobra* |
| | Brita Koivunen | *Suklaasydän* |
| | Tapio Rautavaara | *Isoisän olkihattu* |
| | Kauko Käyhkö | *Rovaniemen markkinoilla* |
| | Georg Ots | *Saarenmaan valssi* |
| | Veikko Tuomi | *Vanhan vaahteran laulu* |
| | Rosemary Clooney | *Mambo italiano* |
| | Pat Boone | *Love Letters in the Sand* |
| | Bill Haley | *Rock Around the Clock* |
| | Elvis Presley | *Heartbreak hotel* |
| | The Four Lads | *Istanbul (Not Constantinople)* |
| | Louis Prima | *Buona Sera* |
| | Glenn Miller | *Moonlight Serenade* |
| 1960s popular songs (30) | Tamara Lund | *Sinun omasi* |
| | Katri Helena | *Minne tuuli kuljettaa* |
| | Reijo Taipale | *Tähdet meren yllä* |
| | Mauno Kuusisto | *Kertokaa se hänelle* |
| | Danny | *Piilopaikka* |
| | Kari Kuuva | *Tango pelargonia* |
| | Four Cats | *Suuret setelit* |
| | Dusty Springfield | *You Don't Have to Say You Love Me* |
| | Billy J. Kramer & The Dakotas | *Bad to Me* |
| | The Monkees | *I'm a Believer* |
| | The Rolling Stones | *The Last Time* |
| | The Animals | *House of the Rising Sun* |
| | The Swinging Blue Jeans | *Hippy Hippy Shake* |
| | Stan Getz & João Gilberto | *The Girl from Ipanema* |
| | Ray Charles | *Hit the Road Jack* |
| | Vieno Kekkonen | *Ei koskaan sunnuntaisin* |
| | Pirkko Mannola | *Kuinka rakkaus alkoi* |
| | Pasi Kaunisto & Nacke Johansson's Orchestra | *Koskaan et muuttua saa* |
| | Eino Grön | *Sä kuulut päivään jokaiseen* |
| | Aikamiehet | *Iltatuulen viesti* |
| | Johnny | *Hyvin menee kuitenkin* |
| | Eero Raittinen | *Vanha holvikirkko* |
| | The Sounds | *Emma* |
| | The Beatles | *All My Loving* |
| | Tom Jones | *Delilah* |

| | | |
|---|---|---|
| | Simon & Garfunkel | *Bridge Over Troubled Water* |
| | Procol Harum | *A Whiter Shade of Pale* |
| | The Beach Boys | *Good Vibrations* |
| | The Renegades | *Cadillac* |
| | Aretha Franklin | *Chain of Fools* |
| **1970s popular songs (30)** | Vicky Rosti | *Tuolta saapuu Charlie Brown* |
| | Merja Rantamäki | *Mistä mä löytäisin sen laulun* |
| | Jukka Kuoppamäki | *Kultaa tai kunniaa* |
| | Jussi & the Boys | *Metsämökin tonttu* |
| | Deep Purple | *Black Night* |
| | Hector | *Olen hautausmaa* |
| | Jamppa Tuominen | *Aamu toi, ilta vei* |
| | Irwin Goodman | *St. Pauli ja Reperbahn* |
| | ABBA | *Waterloo* |
| | Lynn Anderson | *Rose Garden* |
| | Elton John | *Crocodile Rock* |
| | The Rubettes | *Sugar Baby Love* |
| | Creedence Clearwater Revival | *Travelin' Band* |
| | Donna Summer | *Hot Stuff* |
| | Carl Douglas | *Kung Fu Fighting* |
| | Katri Helena | *Syysunelma* |
| | Fredi | *Puhu hiljaa rakkaudesta* |
| | Erkki Junkkarinen | *Ruusuja hopeamaljassa* |
| | Kai Hyttinen | *Dirlanda* |
| | Leevi & the Leavings | *Mitä kuuluu, Marja-Leena?* |
| | Tuomari Nurmio | *Valo yössä* |
| | Hurriganes | *Get on* |
| | Kontra | *Jerry Cotton* |
| | Baccara | *Yes Sir, I Can Boogie* |
| | Middle of the Road | *Chirpy Chirpy Cheep Cheep* |
| | Uriah Heep | *Lady in Black* |
| | Christie | *Yellow River* |
| | Led Zeppelin | *Whole Lotta Love* |
| | Roberta Flack | *Killing Me Softly with His Song* |
| | Boney M. | *Rivers of Babylon* |
| **1980s popular songs (30)** | Paula Koivuniemi | *Tummat silmät, ruskea tukka* |
| | Vera Telenius | *Miljoona ruusua* |
| | Topi Sorsakoski | *Eeva* |
| | Kirka | *Surun pyyhit silmistäni pois* |
| | Pirkka-Pekka Petelius | *Muistan sua Elaine* |
| | Miljoonasade | *Marraskuu* |
| | Leevi & the Leavings | *Pohjois-Karjala* |
| | Pelle Miljoona | *Moottoritie on kuuma* |
| | Barbara Streisand | *Woman in Love* |
| | Madonna | *Papa Don't Preach* |
| | Toto | *Africa* |
| | David Bowie | *Let's Dance* |
| | Earth, Wind & Fire | *Celebration* |
| | Marvin Gaye | *I Heard It through the Grapevine* |
| | Diana Ross | *Upside Down* |
| | Tuula Amberla | *Lulu* |
| | Lea Laurila | *Ei oo, ei tuu* |
| | Matti & Teppo | *Mä näitä polkuja tallaan* |
| | Rauli Badding Somerjoki | *Tähdet tähdet* |
| | Juice Leskinen | *Kaksoiselämää* |
| | J. Karjalainen ja Mustat Lasit | *Doris* |
| | Juha Vainio ja Hyvän Tuulen Laulajat | *Albatrossi* |
| | Eppu Normaali | *Kitara, taivas ja tähdet* |

| | | |
|---|---|---|
| | Blondie | *Call Me* |
| | Michael Jackson | *Billie Jean* |
| | Tina Turner | *Typical Male* |
| | Jari Huhtasalo | *Äideistä parhain* |
| | Leonard Cohen | *Dance Me to the End of Love* |
| | Ottawan | *Hands Up* |
| | Stevie Wonder | *I Just Called to Say I Love You* |
| **Traditional folk songs (20)** | (Trad.) | *Tuoll' on mun kultani* |
| | (Trad.) | *Kalliolle kukkulalle* |
| | (Trad.) | *Tuonne taakse metsämaan* |
| | (Trad.) | *Kotimaani ompi Suomi* |
| | (Trad.) | *Yksi ruusu on kasvanut laaksossa* |
| | (Trad.) | *Leivo* |
| | (Trad.) | *Säkkijärven polkka* |
| | (Trad.) | *Tulatullallaa* |
| | (Trad.) | *On neidolla punapaula* |
| | (Trad.) | *Laulu Suomessa* |
| | (Trad.) | *Taivas on sininen ja valkoinen* |
| | (Trad.) | *Soittajapaimen* |
| | (Trad.) | *Kotini* |
| | (Trad.) | *Lapsuuden toverille* |
| | (Trad.) | *Sunnuntaiaamuna* |
| | (Trad.) | *Jos sais kerran reissullansa* |
| | (Trad.) | *Minun kultani kaunis on* |
| | (Trad.) | *Täällä yksinäni laulelen* |
| | (Trad.) | *On suuri sun rantas autius* |
| | (Trad.) | *Heili Karjalasta* |
