# Supplementary material for: What makes music memorable? Relationships between acoustic musical features and music-evoked emotions and memories in older adults
Source: PLoS One. 2021 May 14;16(5):e0251692. doi: 10.1371/journal.pone.0251692 (PMC8121320; doi:10.1371/journal.pone.0251692)
Supplement: S2 Table — (PDF) [file pone.0251692.s002.pdf]

S2 Table. Bivariate correlations between all musical features.

Feature	AT	F	FC	FE	KC	M	N	PC	RMS	R	SC	SE	SF	SS	SB1	SB2	SB3	SB4	SB5	SB6	SB7	SB8	SB9	SB10
Attack time	1		0.44	-0.47					-0.39				-0.34					-0.51	-0.39					
Flatness		1		-0.37				0.60			**0.87**	0.77	0.39	**0.93**	0.56	0.64	0.5					0.39	0.75	**0.84**
Fluctuation centroid	0.44		1	-0.61	-0.32																			
Fluctuation entropy	-0.47	-0.37	-0.61	1				-0.46			-0.36			-0.34										
Key clarity			-0.32		1					0.31		0.33								0.39				
Mode						1														0.35	0.34			
Novelty							1																	
Pulse clarity		0.60		-0.46				1		0.34	0.59	0.61	0.53	0.49	0.64	0.74	0.54				0.42	0.49	0.55	0.56
RMS energy	-0.39								1	0.60	0.33	0.34	**0.81**		0.44	0.46	0.60	0.58	0.46	0.53	0.60	0.53	0.47	0.44
Roughness					0.31			0.34	0.60	1	0.50	0.59	0.74		0.30	0.32	0.41		0.37	0.62	**0.80**	0.79	0.67	0.53
Spectral centroid		**0.87**		-0.36				0.59	0.33	0.50	1	**0.91**	0.53	**0.89**	0.53	0.62	0.46			0.36	0.47	0.66	**0.92**	**0.88**
Spectral entropy		0.77			0.33			0.61	0.34	0.59	**0.91**	1	0.63	0.70	0.55	0.62	0.48			0.54	0.63	0.74	**0.84**	0.73
Spectral flux	-0.34	0.39						0.53	**0.81**	0.74	0.53	0.63	1	0.36	0.57	0.62	0.71	0.52	0.49	0.75	**0.80**	0.71	0.63	0.58
Spectral spread		**0.93**		-0.34				0.49			**0.89**	0.70	0.36	1	0.50	0.58	0.46					0.35	0.78	**0.86**
Sub-band flux 1		0.56						0.64	0.44	0.30	0.53	0.55	0.57	0.5	1	**0.89**	0.57				0.35	0.43	0.55	0.52
Sub-band flux 2		0.64						0.74	0.46	0.32	0.62	0.62	0.62	0.58	**0.89**	1	0.73				0.39	0.51	0.61	0.58
Sub-band flux 3		0.50						0.54	0.60	0.41	0.46	0.48	0.71	0.46	0.57	0.73	1	0.39			0.39	0.44	0.52	0.48
Sub-band flux 4	-0.51								0.58				0.52				0.39	1	0.59					
Sub-band flux 5	-0.39								0.46	0.37			0.49					0.59	1	0.61	0.37			
Sub-band flux 6					0.39	0.35			0.53	0.62	0.36	0.54	0.75						0.61	1	0.75	0.52	0.43	0.40
Sub-band flux 7						0.34		0.42	0.60	**0.80**	0.47	0.63	**0.80**		0.35	0.39	0.39		0.37	0.75	1	**0.82**	0.53	0.42
Sub-band flux 8		0.39						0.49	0.53	0.79	0.66	0.74	0.71	0.35	0.43	0.51	0.44			0.52	**0.82**	1	0.73	0.51
Sub-band flux 9		**0.75**						0.55	0.47	0.67	**0.92**	**0.84**	0.63	0.78	0.55	0.61	0.52			0.43	0.53	0.73	1	**0.88**
Sub-band flux 10		**0.84**						0.56	0.44	0.53	**0.88**	0.73	0.58	**0.86**	0.52	0.58	0.48			0.40	0.42	0.51	**0.88**	1

Only correlations larger than r = 0.3 are shown and correlations larger than r = 0.8 are shown in bold.
