# Supplementary material for: What makes music memorable? Relationships between acoustic musical features and music-evoked emotions and memories in older adults
Source: PLoS One. 2021 May 14;16(5):e0251692. doi: 10.1371/journal.pone.0251692 (PMC8121320; doi:10.1371/journal.pone.0251692)
Supplement: S3 Table — (PDF) [file pone.0251692.s003.pdf]

S3 Table. Examples songs with highest PCA scores with corresponding musical component and a short description of possible reasons for the high score.

Musical component	Song with highest PCA score	Description
Brightness	Michael Jackson – Billie Jean	Wide sound of snare drum and high pitch guitar riffs.
High-mid	Olavi Virta – Sokeripala	High pitch accordion line.
Pulse strength	Carl Douglas – Kung Fu Fighting	A clear, almost electronic sounding drum beat.
Low-mid	Ottawan – Hands Up	Moving bass line and singing voices at specific pitch area.
Rhythmic clarity	Kauko Käyhkö – Rovaniemen Markkinoilla	Very clear and stabile rhythm in both singing voice and instruments.
Novelty	Led Zeppelin – Whole Lotta Love	Sudden change of instrumentalization.
