# Supplementary material for: What makes music memorable? Relationships between acoustic musical features and music-evoked emotions and memories in older adults
Source: PLoS One. 2021 May 14;16(5):e0251692. doi: 10.1371/journal.pone.0251692 (PMC8121320; doi:10.1371/journal.pone.0251692)
Supplement: S4 Table — (PDF) [file pone.0251692.s004.pdf]

S4 Table. Bivariate correlations, regression coefficients, and p-values of Familiarity mediation models for each emotion variable.

Variables	Familiarity	Emotional intensity	β	p	R^2	F (p)
**Emotional intensity**	.71		0.182	< .0001	.621	55.35 (<.0001)
**Pulse strength**	-.56	-.42	-0.062	< .0001		
**Brightness**	-.31	-.28	-0.030	.003		
**Low-mid**	-.21	-.27	-0.014	.16		
	**Familiarity**	**Valence**	**β**	**p**	**R^2**	**F (p)**
**Valence**	.56		0.126	< .0001	.530	38.03 (<.0001)
**Pulse strength**	-.56	-.33	-0.080	< .0001		
**Brightness**	-.31	-.27	-0.040	.0003		
**Low-mid**	-.21	-.27	-0.023	.04		
	**Familiarity**	**Arousal**	**β**	**p**	**R^2**	**F (p)**
**Arousal**	.47		0.188	< .0001	.557	42.47 (<.0001)
**Pulse strength**	-.56	-.25	-0.085	< .0001		
**Brightness**	-.31	-.04	-0.054	< .0001		
**Low-mid**	-.21	-.01	-0.038	.0002		

β = unstandardized regression coefficient of a single predictor in multiple regression model, after controlling for other variables, p = p-value of a single variable, R^2 = coefficient of determination of the model, F (p) = F-value of the model (p-value of the model in the brackets). Degrees of freedom for all models are 4 and 135.
